# Research on the Fatigue Properties of Rejuvenated Asphalt Prepared by Waste Cooking Oil Pre-Desulfurized Crumb Tire Rubber

**DOI:** 10.3390/polym15030740

**Published:** 2023-01-31

**Authors:** Ruikun Dong, Zhiyu Zhang, Tao Zhou, Weitong Deng, Hong You

**Affiliations:** 1 School of Civil Engineering, Chongqing University, Chongqing 400045, China; 2 Key Laboratory of New Technology for Construction of Cities in Mountain Area, Ministry of Education, Chongqing University, Chongqing 400045, China; 3 Sichuan Communication Surveying & Design Institute Co., Ltd., Chengdu 610017, China

**Keywords:** rejuvenated asphalt, waste tire crumb rubber, waste rubber/oil, fatigue life, evaluation index, dynamic shear rheometer, rheological recoverability

## Abstract

Fatigue cracking has hitherto been a crucial constraint on the development of reclaimed asphalt pavements attributed to the performance of rejuvenated asphalt binder. Therefore, it is extremely significant to evaluate the fatigue performance of rejuvenated asphalt precisely and objectively and to improve the fatigue life of rejuvenated asphalt binders. With preceding research in our group, this paper investigated the fatigue properties of waste rubber/oil (WRO) rejuvenated asphalt and universal rejuvenated asphalt by dynamic shear rheometer test (DSR). The applicability of common fatigue life evaluation indexes and the response to internal and external influences on the fatigue performance of rejuvenated asphalt were analyzed. It is demonstrated that N*_p20_* corresponding to the mutagenesis of phase angle is physically significant and independent of the parameters including rejuvenator type, loading mode and loading level, which was recommended as the evaluation index for fatigue life of rejuvenated asphalt in this paper. The fatigue performance of both WRO and universal rejuvenated asphalt is found to decrease with loading frequency and loading level, but the fatigue life of WRO rejuvenated asphalt is comparatively superior to the latter, particularly at high loading frequencies and levels. Influenced by waste tire crumb rubber (WTCR), increasing the proportion of WTCR can improve the fatigue life of rejuvenated asphalt. When compared to other rejuvenated asphalt, RWRO@55 rejuvenated asphalt shows better fatigue performance and its fatigue life rebounds at high loading frequency. Consequently, the recommended mastic–oil ratio is 5:5. However, when the rheological recoverability compensation is considered, the fatigue lifetime evaluation of rejuvenated asphalt will be changed significantly, and therefore the fatigue performance evaluation of rejuvenated asphalt should consider the influence of rheological recoverability to develop a comprehensive evaluation system.

## 1. Introduction

The utilization of reclaimed asphalt pavement (RAP) provides a particularly preferable alternative to the construction of asphalt pavements due to the significant amount of aggregate and asphalt binder contained in RAP material, miraculously saving an abundant number of natural and financial resources, which is environmentally friendly and economical [1]. However, rejuvenated asphalt pavement is predisposed to fatigue cracking, and its fatigue life is generally associated with the properties of both mixture and asphalt binder, and the gradation and environmental factors [2,3,4]. According to the research of French Laboratoire Central des Ponts et Chaussées (LCPC), the fatigue failure of asphalt mixtures was predominantly caused by cracking or damage to asphalt binder [5]. Concurrently, it was concluded by the Strategic Highway Research Program (SHRP) that asphalt contributed 52% to the fatigue performance of asphalt mixtures [6,7]. Therefore, investigating the fatigue life of asphalt binders is of positive significance for further research into the fatigue performance of asphalt mixtures.

The fatigue performance of asphalt binders can be significantly influenced by external loadings and admixtures. Moreover, there will be rheological rehabilitation internally in asphalt due to its viscoelastic characteristics, which consequently makes a difference to the fatigue performance. To ameliorate the stiffening properties of an aged binder, its rheological properties should be rehabilitated with a rejuvenator or gelatinous asphalt [8,9]. Therefore, scholars have been passionately devoted to the research of rejuvenators to develop rejuvenated asphalt with excellent performance. Yazdipanah [10], Yi [11,12], and Dong [13,14,15,16] have found that degradation of waste tire crumb rubber (WCTR) in waste cooking oil (WCO) can provide more prominent activation of waste rubber/oil rejuvenator (WRO), where WRO as a modifier or raw material incorporated into matrix asphalt or aged asphalt can improve the performance of asphalt. The crumb rubber and waste frying oil can restore the properties of RAP asphalt, especially its rheological properties, and also improve the compatibility and storage stability of RAP binder [17]. It was identified by Bilema et al. [18] that waste frying oil could restore the mechanical properties such as the tensile strength ratio of recycled asphalt pavement, and crumb rubber could improve the rutting resistance of asphalt mixture, which are complementary to each other. Additionally, when pretreated with WCO at high temperatures, the three-dimensional network structure in crumb rubber is swollen and the interaction between rubber chains is weakened, which consequently leads to cross-linked bonds such as S-S and S-C with comparatively low bond energy being broken, and the soluble linear rubber molecular chains, carbon black fragments and other medium and small molecular weight substances are released [16,19]. The released carbon black can inhibit the conversion of saturates and reduce the consumption of soft asphalt, which can significantly improve the mid-temperature fatigue performance of asphalt [20,21]. It can be seen that the waste cooking oil pre-desulfurized crumb tire rubber is supplementary to the elasticity of asphalt, and can improve the rheological properties of rejuvenated asphalt, with significant amounts of light components, carbon black and other products released simultaneously, all of which make a positive contribution to the fatigue performance improvement of rejuvenated asphalt. Therefore, there is a significant need to explore the fatigue performance of rejuvenated asphalt prepared by waste cooking oil and crumb tire rubber in comparison to that of commonly commercial rejuvenators, and to analyze the effect of WRO and WTCR dosage on the fatigue performance of rejuvenated asphalt, as this is where it differentiates itself from other rejuvenators.

Since the fatigue performance of asphalt binders is considered to be important to the service life of rejuvenated asphalt pavements, many scholars have exhaustively examined the evaluation indexes on fatigue life of asphalt binder. It was supposed persuasively by Liao [22,23] and Wen et al. [24] that the complex modulus of asphalt binder would decrease with loading times during the fatigue damage stage, and the number of loading times corresponding to a reduction of the complex modulus to 50% of its initial modulus was proposed as the fatigue life (N*_f50_*) of asphalt binder. Based on a Simplified Viscoelastic Continuum Damage (S-VECD) model and Linear Amplitude Sweep (LAS) test, the damage at fatigue failure of asphalt was defined by Chen [25] as the accumulated damage equivalent to a 35% reduction in undamaged value of $\left|G^{*}\right|sin\delta$. Cao [26] concluded that when asphalt binders were loaded under stress-controlled mode, the changing rate of the complex modulus gradually deviated from the initial value as the loading times increased, with an inflection point appearing, which was defined as the fatigue life (N_RG*_) of the asphalt binder. It has also been recommended that the fatigue performance of asphalt can be evaluated by a dissipative energy ratio (DER) [27,28,29,30,31,32,33,34]. Pronk [29] presented the cumulative dissipation energy ratio (CDER) as a basis to determine the fatigue performance of asphalt binder when considering the influence of loading history. Bonnetti [30] defined that the loading times corresponding to a pivotal point where CDER curve deviated from the nondestructive curve by 20% as the fatigue life of asphalt. Additionally, the rate of dissipated energy change (RDEC) was developed by Carpenter [31,32] and Margaritis et al. [33] to evaluate the fatigue performance and damage state of asphalt binder. It was proved by Pitawala et al. [34] that RDEC is a useful method to evaluate the dissipated energy (DE) variation of asphalt. However, all of the evaluation indexes mentioned above are mechanically and energy based, of which the appropriateness to evaluate the fatigue performance of rejuvenated asphalt needs to be discussed when considering the complexity of the composition and properties of WRO rejuvenated asphalt.

In this paper, the fatigue life evaluation indexes and major influencing factors of rejuvenated asphalt are studied to evaluate the fatigue performance of WRO rejuvenated asphalt (RWRO) objectively, which is important to improve the fatigue and service life of recycled asphalt pavements and the recycling of waste pollutants such as RAP, WCO and WTCR. Firstly, fatigue tests were conducted on rejuvenated asphalt under different loading modes and loading levels by dynamic shear rheometer (DSR), by which the applicability of common fatigue life evaluation and internal and external influencing factors were analyzed. In addition, the effect of rheological recovery on fatigue performance was investigated based on a rheological recovery test. More importantly, the fatigue properties of RWRO and EVO rejuvenated asphalt (REVO, universal rejuvenated asphalt) were compared.

## 2. Materials and Methods

### 2.1. Materials

#### 2.1.1. Asphalt, WTCR and WCO

The properties of aged asphalt obtained in RAP are susceptible to the effects of extracts, which may affect the performance of rejuvenated asphalt and test results. To obtain aged asphalt with stable performance, artificially aged asphalt was chosen rather than extracted RAP binder in this study [11,35,36]. One 70# matrix asphalt was aged at 163 °C for 85 min through a standard Rolling Thin Film Oven (RTFO, SHANGHAI CHANGJI GEOLOGICAL Inc., Shanghai, China) to obtain short-term aging asphalt (70# RT) according to AASHTO T240. Furthermore, the 70#RT was aged for 20 h by pressure aging vessel (PAV, Prentex Inc., Dallas, TX, USA) at 100 °C and 2.1 mpa to obtain long-term aging asphalt (70#PAV) according to AASHTO R28. The primary technical specifications of matrix asphalt and aged asphalt are shown in Table 1, and the detailed component of WTCR, a 40-mesh radial tire, is presented in Table 2. The WTCR was a 40-mesh radial tire, and the component of WTCR is presented in Table 2. The fundamental characteristics and major aliphatic acid contents of WCO utilized in this paper are listed in Table 3.

#### 2.1.2. Preparation of WRO Rejuvenator

**Figure 1:**
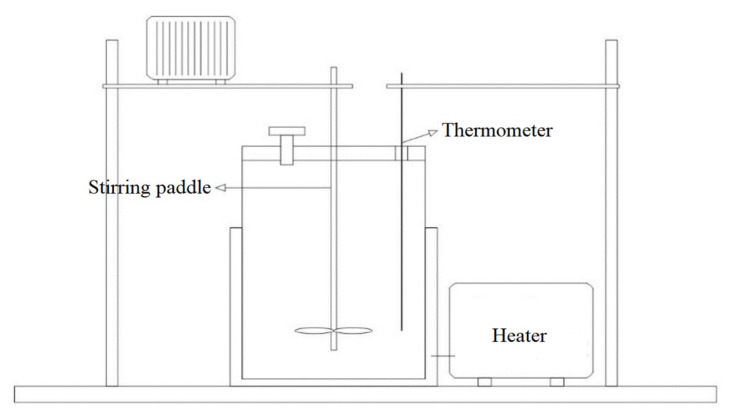
Brief diagram of laboratory self-made reaction device.

The WTCR and WCO were accurately weighed in mass ratios (3:7, 4:6, and 5:5), homogenized and then placed at room temperature for 2 h based on the research of our group [11,12,13,14,15,16]. Subsequently, the mixture was put into the reaction device (Figure 1) and heated to 260 °C ± 10 °C at 8 °C/min with continuous insulation for 1 h. The stirring speed was 250–300 rpm. Additionally, the preparation process was proceeded at standard atmospheric pressure, where the atmosphere in the reaction apparatus was air.

#### 2.1.3. Preparation of Rejuvenated Asphalt

The 70#PAV was melted in an oven at 150 °C and gently poured into the heating device. Then, the rejuvenators (Two commercial rejuvenators, Evoflex@8182 and Evoflex@ca3, and three WRO rejuvenators, of which the rubber/oil ratio was 5:5, 3:7 and 4:6, named WRO@55, WRO@37 and WRO@46, respectively.) were added to the 70#PAVwith a content of 6 wt.%, 8 wt.%, and 10 wt.%, respectively. Ultimately, all mixtures were heated for 0.5~1 h with a stirring speed of 300~350 rpm at 160 ± 5 °C. The asphalt samples utilized in this research are detailed in Table 4 and the basic technical indexes of rejuvenated asphalt (8% rejuvenator) are shown in Table 5.

### 2.2. Test Methods

#### 2.2.1. Fatigue Test of Rejuvenated Asphalt

In this study, a time-sweep (TS) test was performed with Dynamic Shear Rheometer (DSR, AR 1500 ex Instrument, TA Co., New Castle, DE, USA) to evaluate the fatigue properties of rejuvenated asphalt (10% rejuvenator). The test was conducted in the stress-controlled mode of 4.5%, 5.5% and 6.5% or strain-controlled mode of 100 kpa with an 8 mm-diameter parallel plate geometry (2 mm gap). The load frequencies were 5 Hz, 10 Hz, and 15 Hz and the test temperature was 20 °C.

Depending on the TS test, the mechanical properties of rejuvenated asphalt will decrease, which is mainly manifested by a sustained decrease of complex modulus (G*) and a sustained increase of phase angle (δ). Consequently, the fatigue behavior of asphalt has been characterized from a phenomenological and energetic perspective by relevant scholars, as shown in Table 6.

#### 2.2.2. Rheological Recovery Test of Rejuvenated Asphalt

The variation in complex modulus of rejuvenated asphalt is implemented to characterize the restoration of rheological properties. The test was primarily performed by Dynamic Shear Rheometer (DSR, AR 1500 ex Instrument, TA Co., New Castle, DE, USA) in strain-controlled mode at 4.5% with an 8 mm-diameter parallel plate geometry (2 mm gap). The load frequency was 10 Hz, and the test temperature was 20 °C.

**Figure 2:**
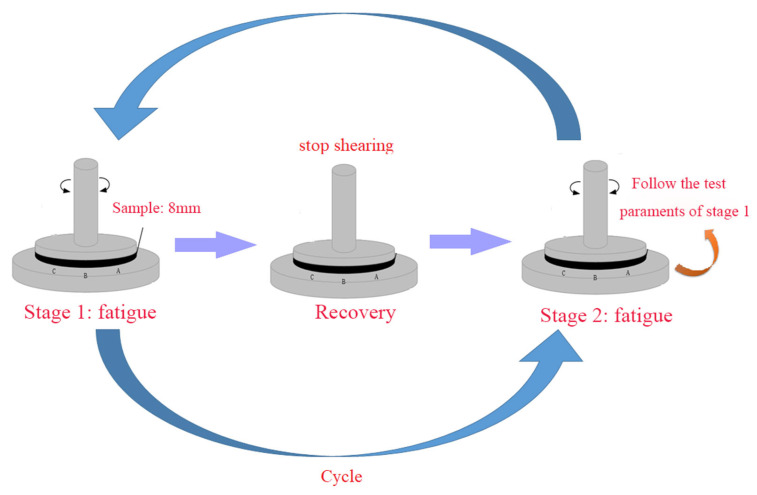
Rheological recovery test method.

The rheological recovery test was conducted in the fatigue–healing–fatigue test mode: the first stage was promptly stopped when the complex modulus dropped to 40% initial complex modulus; then, the damaged asphalt was heated to different temperatures (30 °C, 50 °C, 70 °C), which were subsequently healed for 5 min, 10 min, 20 min, and 40 min; ultimately, the rejuvenated asphalt was cooled to 20 °C and subjected to the original fatigue parameters for rheological recovery test (Figure 2).

Furthermore, the effect of rheological recovery properties of rejuvenated asphalt on fatigue life is analyzed by the recovery degree of fatigue life (*RF*).

(1) $$RF=Nf_{a}/Nf_{b}$$

where, *RF* is the recovery degree of fatigue life; $Nf_{a}$ is the fatigue life after healing; and $Nf_{b}$ is the fatigue life before healing.

## 3. Results and Discussion

### 3.1. Analysis of Common Fatigue Performance Evaluation Indexes

#### 3.1.1. Applicability Analysis of Common Evaluation Indicators

The fatigue lifetimes of RWRO and REVO are evaluated in this section based on the above indicators (Table 6), as shown in Table 7, Table 8 and Table 9, where the applicability of repeatedly used evaluation indicators is further discussed to determine the appropriate fatigue lifetime evaluation indicators.

**Figure 3:**
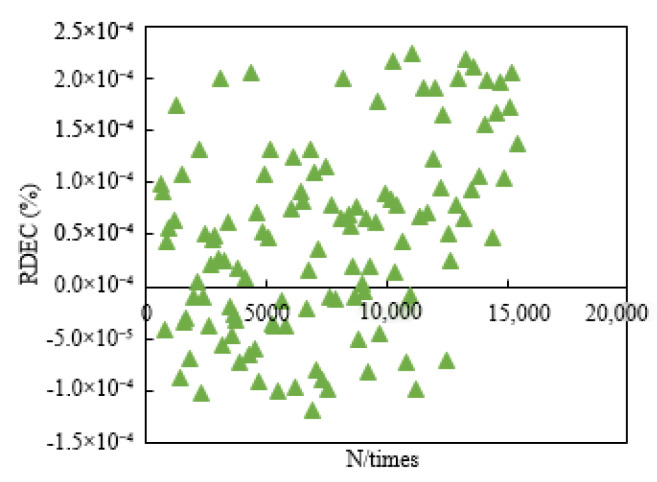
Evaluation result of N*_fm_* under strain-control.

**Figure 4:**
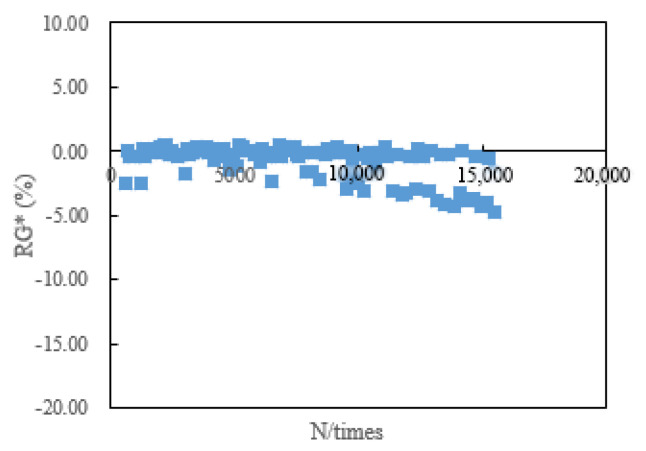
Evaluation result of N_RG*_ under strain-control.

The fatigue life of RWRO is better than that of REVO according to Table 7, Table 8 and Table 9. Simultaneously, N_RG*_ and N*_fm_* cannot evaluate the fatigue life of rejuvenated asphalt in the strain-controlled mode. It is revealed in Figure 3 that N*_fm_* is influenced by the loading mode with significant discretization when adopting the strain-controlled loading mode, which cannot evaluate the fatigue life of rejuvenated asphalt and was also proven by Wen et al. [24]. The change rate of complex modulus (RG*) oscillates in the strain-control mode without obvious mutations according to Figure 4, which makes it impracticable to determine the fatigue life of rejuvenated asphalt. It is attributed to the changes in the components of asphalt after aging, which are manifested as a decrease in the aromatic content and an increase in the asphaltene content, causing a hardening of the whole asphalt colloidal structure and an increase in the complex modulus [17]. Although part of the aged asphalt can be softened by rejuvenators, the rejuvenated asphalt contains some aged components that can result in strong resistance to deformation, and the dissipated energy does not change significantly in the strain-controlled mode, instead appearing as a minor change. In addition, the increased complex modulus can provide resistance to the decay rate of the modulus, making no significant changes in complex modulus between adjacent loading times in the strain-controlled mode.

**Figure 5:**
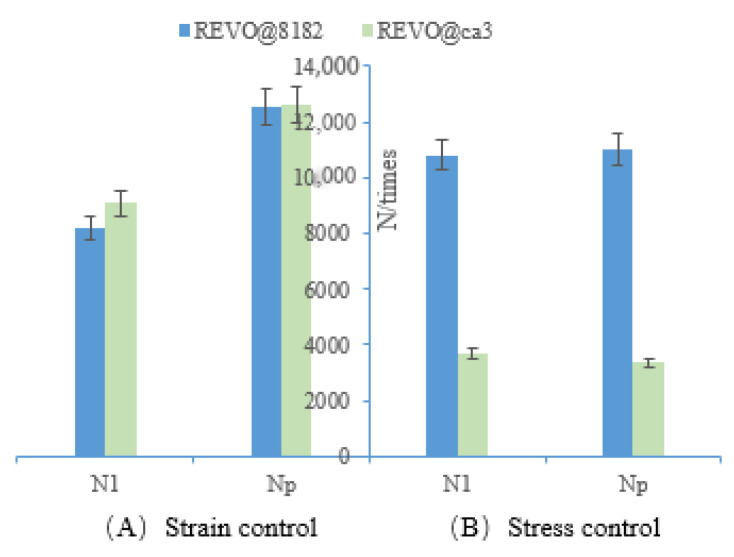
Evaluation results of N*_1_* and N*_p._* under stress (100 kpa)/strain (4.5%) mode.

**Figure 6:**
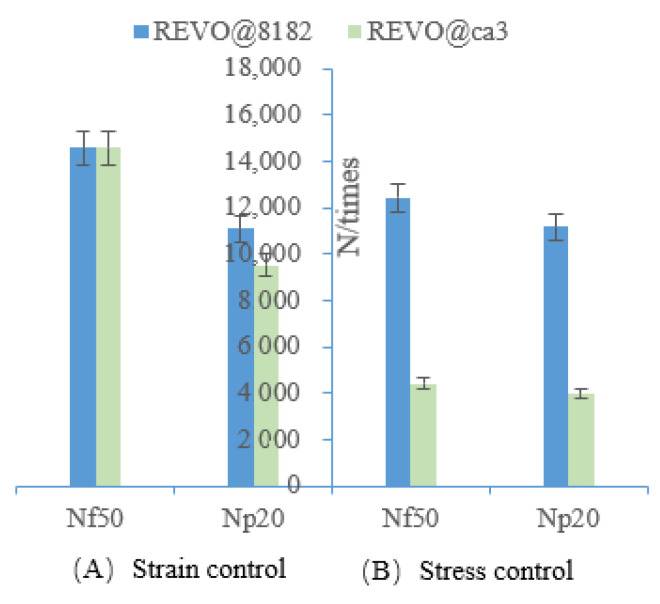
Evaluation results of N*_f50_* and N*_p20._* under stress (100 kpa)/strain (4.5%) mode.

Furthermore, it can be convincingly demonstrated from Table 7, Table 8 and Table 9 that the evaluation results of N*_1_* and N*_p_* are not consistent at different strain levels, with susceptibility to the influence of loading pattern and rejuvenator type. When the strain-controlled levels are 4.5% and 5.5%, the evaluation results of N*_f50_* and N*_p20_* show that the fatigue life of REVO@8182 is better than that of REVO@CA3, which, however, is completely opposite to the evaluation results for N*_1_* and N*_p_*. However, when the strain is controlled at 6.5%, the evaluation results for N*_f50_*, N*_1_*, N*_p_* and N*_p20_* are convergent, i.e., the fatigue life of REVO@8182 is better than that of REVO@CA3. It is indicated that the evaluation results of N_1_ and N*_p_* are susceptible to the influence of load level. Additionally, the evaluation results for RWRO@55, RWRO@37 and RWRO@46 are consistent under various loading modes, without being influenced by loading levels, which suggests that the evaluation results of N*_1_* and N*_p_* are dependent on the rejuvenator type. Additionally, the fatigue life evaluation results of REVO@8182 and REVO@CA3 are completely opposite under stress-controlled and strain-controlled modes (Figure 5), while those of N*_f50_* and N*_p20_* are consistent (Figure 6), indicating that N*_1_* and N*_p_* are equally affected by the loading mode.

Through the preceding analysis, the following conclusions can be found.

- N_RG*_ and N*_fm_* cannot evaluate the fatigue life of rejuvenated asphalt under the strain-control mode;
- N*_1_* and N*_P_* are definitely affected by the strain level, loading model, and the type of rejuvenator, which makes it unavailable to evaluate the effectiveness of the results;
- N*_f50_* and N*_p20_* are independent of loading mode and strain level, so they can be recommended as the evaluation indicators for fatigue life of rejuvenated asphalt.

#### 3.1.2. Correlation Analysis of N_f50_ and N_p20_

**Figure 7:**
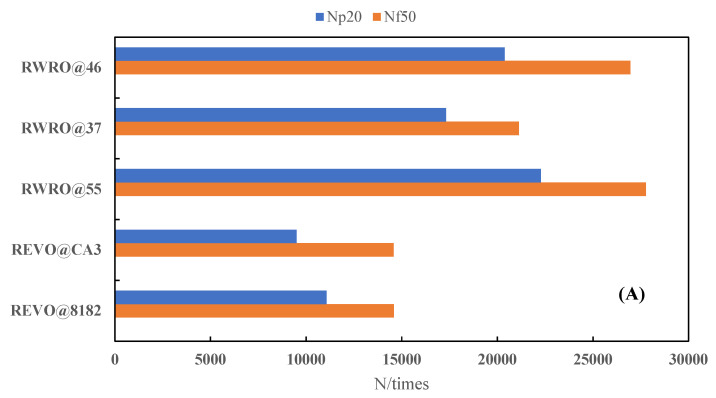

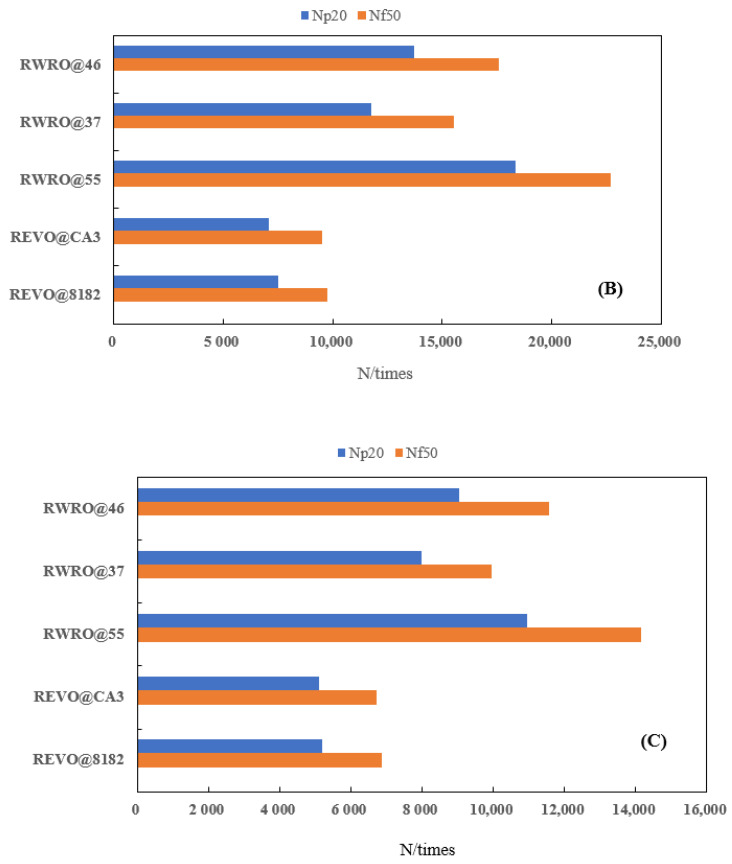
(**A**) Fatigue life of rejuvenated asphalt under 4.5% strain-control. (**B**) Fatigue life of rejuvenated asphalt under 5.5% strain-control. (**C**) Fatigue life of rejuvenated asphalt under 6.5% straincontrol.

**Figure 8:**
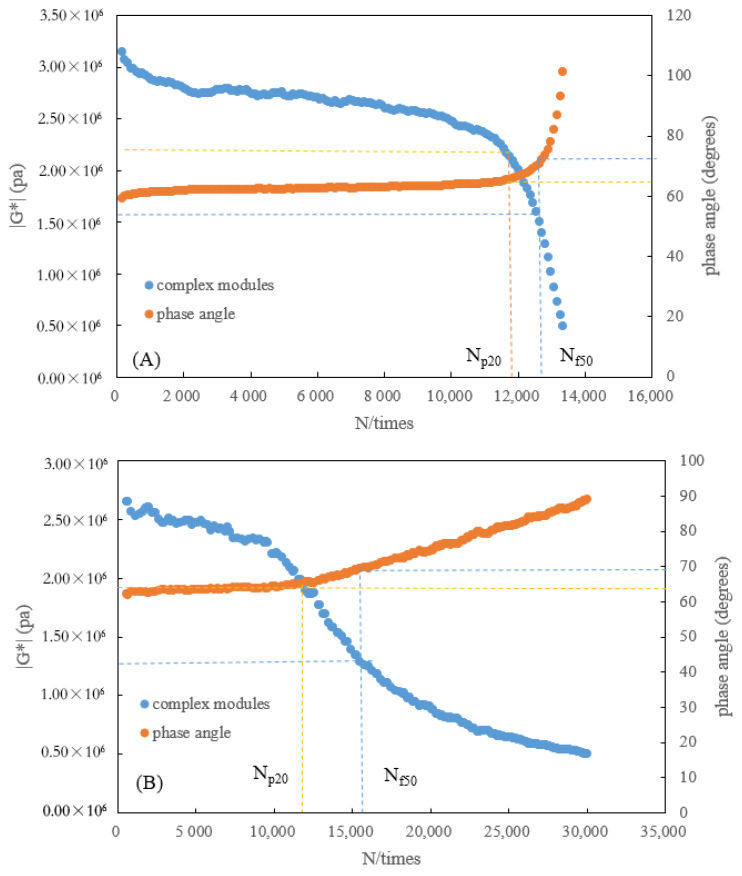
(**A**) Phase angle and complex modulus corresponding to N*_p20_* and N*_f50_* under stress-control. (**B**) Phase angle and complex modulus corresponding to N*_p20_* and N*_f50_* under strain-control.

**Figure 9:**
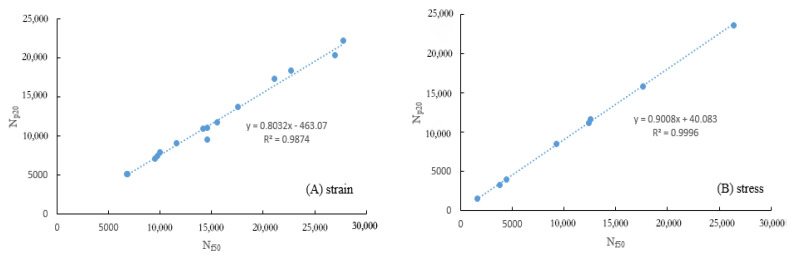
Correlation between N*_p20_* and N*_f50_* under strain/stress-control.

The fatigue life evaluated with N*_f50_* is higher than that with N*_p20_* at all different strain levels, pursuant to Figure 7. This is because N*_f50_* and N*_p20_* correspond to different damage stages, with N*_f50_* representing the “ultimate” fatigue life of asphalt, where macroscopic cracks emerge and accelerate until the specimen is destabilized and fails completely, and N*_p20_* corresponding to the “yield” fatigue life of asphalt where microcracks develop rapidly and cumulatively. Additionally, these conclusions can be verified by comparing the complex modulus and phase angle associated with the assessment of N*_f50_* and N*_p20_* (Figure 8). However, Wen et al. [24] argued that the geometric failure location of N*_f50_* corresponded to the sharp attenuation stage of fatigue damage, where the modified asphalt was approaching a state of complete fatigue damage. The phase angle corresponding to N*_p20_* is smaller than that of N*_f50_*, and the complex modulus for N*_p20_* is larger than that of N*_f50_*, indicating that there is less damage to asphalt with N*_p20_*. Simultaneously, the phase angle that corresponds to N*_p20_* is located at the inflection point of accelerated change in phase angle, with significant physical implications, which is consistent with the conclusion that the loading time corresponding to the inflection point of the phase angle change was taken as the fatigue life of asphalt in a related study [38]. Furthermore, the relationship between N*_p20_* and N*_f50_* is illustrated in Figure 9. There is a well-established linear relationship between N*_p20_* and N*_f50_* (R^2^ greater than 0.95), with the slope of the regression line approximating 1, which suggests that the two fatigue life evaluation indicators are consistent under an identical experimental environment.

Based on the above analysis, N*_f50_* and N*_p20_* can both be used as the evaluation index of fatigue life of rejuvenated asphalt, but the physical significance and the damage state are significantly different. Additionally, the rejuvenated asphalt still contains some aged components, and the fundamental differences in compositions can lead to differences in viscoelasticity, which affects the deformation resistance of the material. It is worthwhile to examine whether the fatigue performance of rejuvenated asphalt can be authentically reflected by exploiting the decay of asphalt complex modulus to 50% of its initial value to determine the fatigue life. Therefore, in this paper, N*_p20_* is recommended as the evaluation index for fatigue life of rejuvenated asphalt, with its specific physical significance and fatigue state.

### 3.2. Influence of Loading Frequency and Loading Level on Fatigue Life of Rejuvenated Asphalt

#### 3.2.1. Loading Frequency

**Figure 10:**
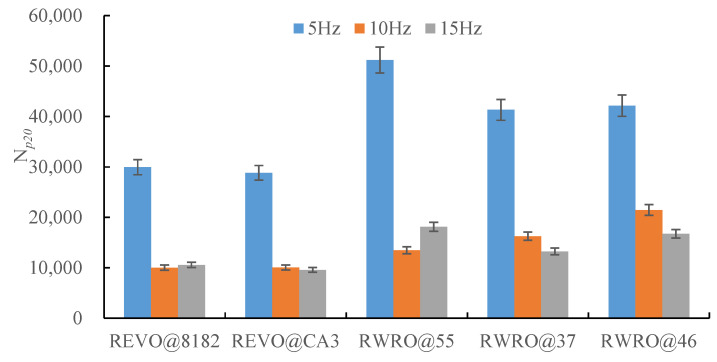
Fatigue life of rejuvenated asphalt under different loading frequencies.

Figure 10 demonstrates the fatigue life of each rejuvenated asphalt at different loading frequencies. When the initial loading frequency is 5 Hz, each rejuvenated asphalt shows excellent fatigue resistance. However, the fatigue life of each rejuvenated asphalt decreases significantly with the increase in loading frequency, which was consistent with Liao’s findings [22,23]. According to Table 10, the fatigue life of REVO@8182, REVO@CA3, RWRO@55 and RWRO@37 decreases substantially when the loading frequency increases to 10 Hz, with an attenuation of more than 60%. Furthermore, when the loading frequency is increased to 15 Hz, the fatigue life of REVO@CA3, RWRO@37 and RWRO@46 continues to decrease, while that of RWRO@8182 and RWRO@55 keeps stable or rebounds slightly, indicating that the latter still has good fatigue performance under high-frequency loading. Moreover, it is demonstrated that RWRO can withstand high-frequency loading since its fatigue life is better than that of REVO at an identical loading frequency.

The fatigue capacity of RWRO will be changed by the ratio of rubber to oil. It can be observed that the fatigue life of RWRO@55 with more rubber does not consistently decrease with increasing loading frequency. This is because the inclusion of more WTCR can prevent the further development of cracks and increase the elastomeric component of colloids, which enhances its elastic recovery and improves its fatigue performance [15,19]. Furthermore, when comparing RWRO with a mastic–oil ratio of 3:7, 4:6 and 5:5, it is revealed that the overall fatigue life of RWRO increases with mastic–oil ratio under identical external loading conditions, which indicates that increasing the ratio of rubber to oil is beneficial to improve the fatigue life of rejuvenated asphalt. However, the ratio cannot be increased indefinitely, and the diffusion capacity of WRO and other properties of rejuvenated asphalt should be considered.

#### 3.2.2. Loading Level

**Figure 11:**
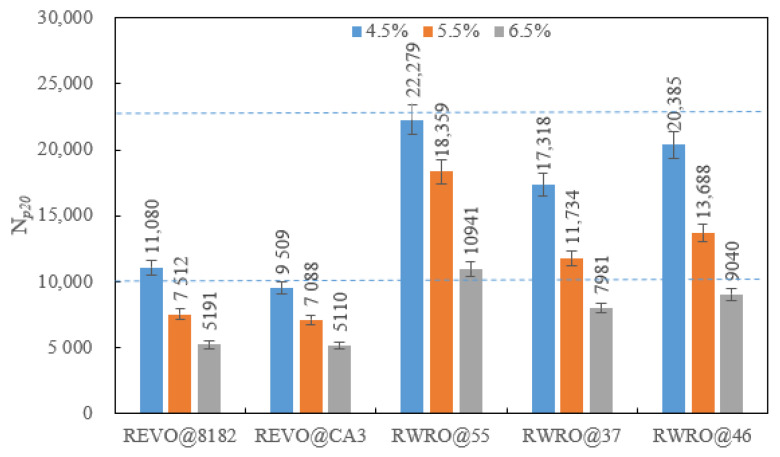
Fatigue life of rejuvenated asphalt under different loading level.

Figure 11 demonstrates the fatigue life of each rejuvenated asphalt at diverse loading levels. It can be recognized that the fatigue life of each rejuvenated asphalt decreases with the loading level under a strain-controlled mode. However, the response of rejuvenated asphalt to loading level is quite different because of the properties of rejuvenator. The N*_p20_* of RWRO under each loading level is higher than that of REVO, which means RWRO with higher rubber content exhibits a better fatigue lifetime. In addition, the change rate of fatigue lives for RWRO and REVO are at an identical level based on Table 11, which indicates that RWRO and REVO are similar in fatigue sensitivity, but there is a significant discrepancy between their loading capacities.

**Figure 12:**
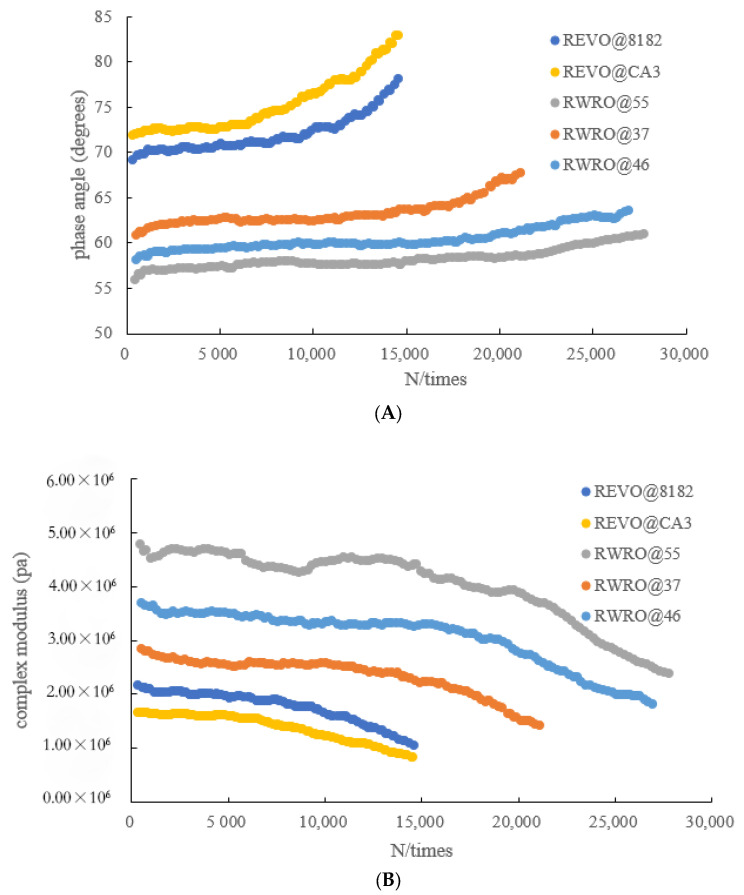
(**A**) Relationship between phase angle and loading times. (**B**) Relationship between phase angle and loading times.

To analyze the reasons for the above conclusions, rheological indicators are introduced in this paper. The initial phase angle and change rate of phase angle of RWRO are less than those of REVO, and its initial complex module is greater than that of REVO, as shown in Figure 12, indicating that RWRO shows improved resistance to deformation, which contributes to improving the fatigue life of rejuvenated asphalt. Therefore, WRO rejuvenator also provides modified effectiveness when compared with commercial rejuvenators, in which the WTCR component can improve the elastomeric recoverability and crack resistance of rejuvenated asphalt, lowering the impact of loading levels on the fatigue life of rejuvenated asphalt and guaranteeing that RWRO can withstand a higher loading level. In addition, when change rate of the phase angle is larger or lower, it can be ensured that the rejuvenated asphalt is minimally affected by loading levels according to Table 11 and Figure 12A. This is because a larger change rate of phase angle can promote a better flowability of the whole rejuvenated asphalt colloid, which may facilitate the self-healing of microcracks and alleviate the effect of increasing loading levels on its fatigue life. When the change rate of the phase angle is smaller, an increase in the elastic components is achieved, which can strengthen the resistance to deformation and also mitigate the effect of loading levels.

### 3.3. Influence of Rejuvenator and Rheological Recovery on Fatigue Life of Rejuvenated Asphalt

#### 3.3.1. Rejuvenator

**Figure 13:**
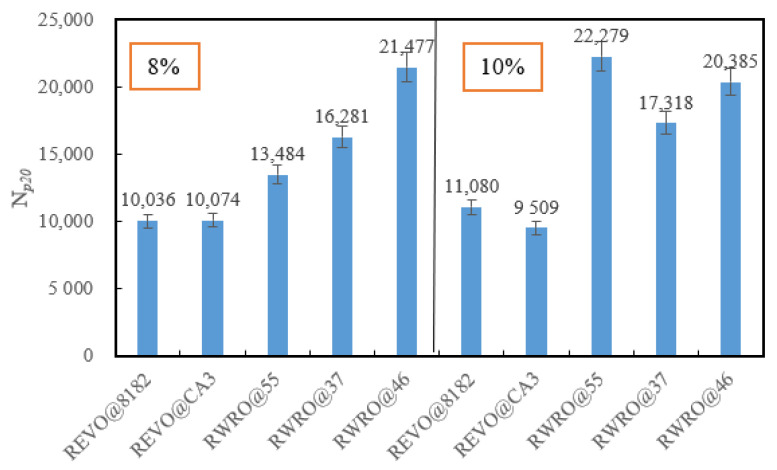
Fatigue life of rejuvenated asphalt.

Figure 13 demonstrates the fatigue life of RWRO and RWEO at different rejuvenator content levels. When the rejuvenator content is 8%, the best fatigue life is achieved with RWRO@46, while REVO@8182 is the worst. Instead, when the content reaches 10%, RWRO@55 shows the best fatigue life, with REVO@CA3 being the worst. This shows that the fatigue life of RWRO is higher than that of RWEO, irrespective of whether the rejuvenator content is 8% or 10%, which indicates that WRO rejuvenator can provide excellent fatigue modification capability that increases significantly with the rejuvenator contents and the ratios of rubber to oil. Furthermore, the fatigue life of REVO@CA3 and RWRO@46 decreases as the content of the rejuvenator increases, demonstrating that their rejuvenator content should not exceed 8%.

#### 3.3.2. Rheological Recovery

**Figure 14:**
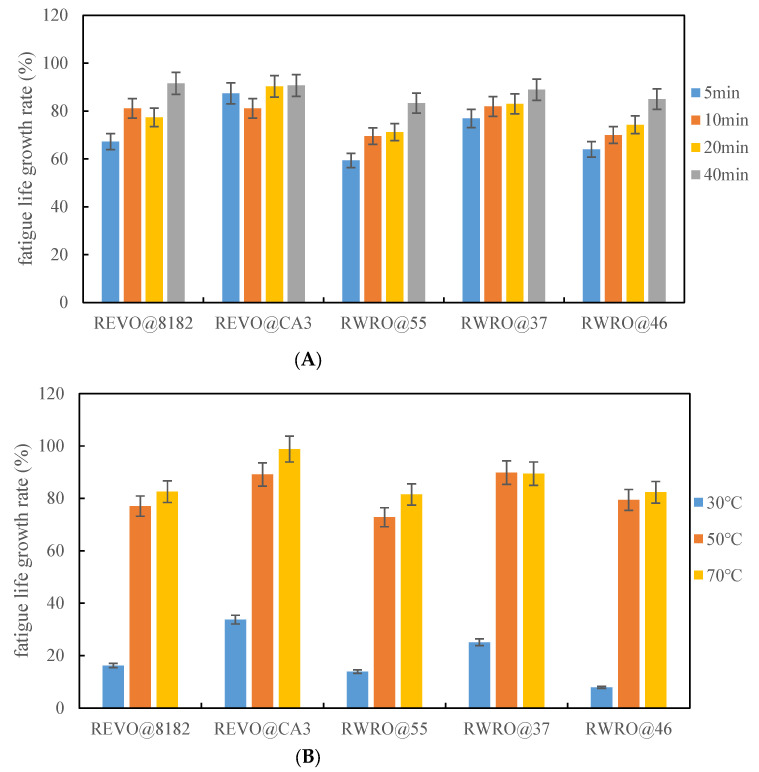
(**A**) Effect of healing time on fatigue life of rejuvenated asphalt. (**B**) Effect of healing temperature on fatigue life of rejuvenated asphalt.

With extensive research, many scholars [39,40,41,42,43,44,45] have discovered that, unlike completely elastomeric materials, the characteristics of asphalt like viscoelasticity, wettability, diffusivity and fluidity can promote its self-healing under certain conditions, typically manifested as asphalt rheological recovery. Therefore, the fatigue performance of rejuvenated asphalt is also affected by rheological recovery. To evaluate the fatigue performance of rejuvenated asphalt comprehensively, this paper explored the influence of rheological recovery on the fatigue life of rejuvenated asphalt. Figure 14 presents the recovery degree of fatigue life for each rejuvenated asphalt under different healing conditions.

It is found that an interval after the cessation of external loading can promote the fatigue life recovery of rejuvenated asphalt, which also proves the availability of rheological recovery. More significantly, the recovery degree of fatigue life for RWRO increases continuously with healing time, reaching more than 80%. Although the recovery degree of fatigue life is oscillating upwards for REVO, it can also be assumed that the fatigue performance of rejuvenated asphalt can be further recovered by prolonging the healing time. Furthermore, the viscosity and plasticity of asphalt will change with temperature, and the activation energy of viscous flow will also change when the temperature increases, which means that the rheological and fatigue recoverability of asphalt is temperature-dependent. As can be noticed from Figure 14B, the recovery degree of fatigue life is generally lower when the initial healing temperature is 30 °C, suggesting a lower healing temperature cannot effectively promote the recovery of fatigue life of rejuvenated asphalt. When the temperature reaches 50 °C, the recovery degree of fatigue life increases greatly, by more than 70%. However, when the temperature is further increased to 70 °C, the recovery degree of fatigue life is no longer significantly improved, or even decreases, indicating that the healing temperature cannot be excessive. Once the temperature limitation where the rheological diffusion of rejuvenated asphalt is exceeded, the recoverability will be seriously affected.

**Figure 15:**
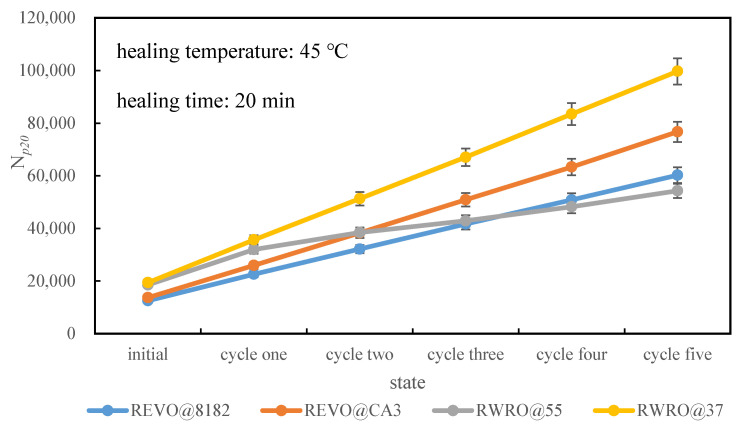
Fatigue life considering self-heling compensation.

Figure 15 demonstrates the fatigue life of each rejuvenated asphalt when considering rheological recovery compensation. When the rheological recovery of rejuvenated asphalt is unconsidered, RWRO@37 shows the best fatigue life, while REVO@8182 is the worst. However, there is a change in the evaluation results when the rheological recovery compensation is considered, where the fatigue life of rejuvenated asphalt with RWRO@37 remains the best, while that with RWRO@55 becomes the worst, with the evaluation results dissimilar to the initial ones after five rheological restoration cycles.

Therefore, with the foregoing analysis, it is essential to comprehensively consider the influence of rheological recovery when evaluating the fatigue life of rejuvenated asphalt, making the evaluation results more compatible with the practical circumstances of rejuvenated asphalt pavement.

## 4. Conclusions

In this paper, the fatigue loading of rejuvenated asphalt was performed with DSR, and the applicability of commonly used fatigue life evaluation indexes was discussed. Additionally, the fatigue properties of RWRO and REVO were analyzed, coupled with the response to internal and external influencing factors based on our previous research. The conclusions are as follows:

(1) N*_p20_* and N*_f50_* are unaffected by the type of rejuvenator, loading mode and loading level, with excellent objectivity and applicability, and the correlation between the two indexes is obvious. Moreover, N*_p20_* corresponds to the mutagenesis of phase angle and microcrack development point, which possesses good physical significance. In this paper, N*_p20_* is recommended as the fatigue life evaluation index for rejuvenated asphalt.

(2) The fatigue life of rejuvenated asphalt decreases with loading frequency and loading level, but the WRO rejuvenator shows better fatigue modification ability. The fatigue life of rejuvenated asphalt prepared by WRO rejuvenator is better than that of REVO, particularly at high loading frequencies and loading levels.

(3) The overall fatigue life of RWRO rejuvenated asphalt increases with the mastic–oil ratio. Additionally, there is no continuous decrease in fatigue life of RWRO@55 as loading frequency increases, with a slight increase occurring at high loading frequency. Consequently, the optimum mastic–oil ratio recommended in this paper is 5:5.

(4) The fatigue life of rejuvenated asphalt can be restored under certain conditions, but the healing temperature should be controlled. The fatigue recovery effect of RWRO at an initial healing temperature of 50 °C can achieve that of REVO. Meanwhile, the fatigue performance evaluation of rejuvenated asphalt needs to consider the influence of rheological recovery and establish an evaluation system considering rheological recovery.

## Figures and Tables

**Table 1:** Test results of matrix asphalt and aged asphalt.

Property	ASTM Standard	Value for		
		70#	70#RT	70#PAV
Softening point/°C	D36	46.6	49.5	57.2
Penetration at 25 °C/(0.1 mm)	D5	73.4	35.6	24.9

**Table 2:** Component of WTCR.

Operating Oil (wt.%)	Rubber Hydrocarbon (wt.%)	Carbon Black (wt.%)	Mineral Filler (wt.%)
5.92	53.54	31.20	9.34

**Table 3:** Main physical and chemical properties of WCO.

Flashpoint (°C)	Saturated Fatty Acid (wt.%)	Unsaturated Fatty Acid (wt.%)
302	18.45	81.55

**Table 4:** The asphalt samples utilized in this research.

Samples	Content (WTCR: WCO)	Attribute	Mix Design of Asphalt Binders
REVO@8182	——	Control group	EVO@8182+70#PAV
REVO@CA3			EVO@CA3+70#PAV
RWRO@55	5:5	——	WRO@55+70#PAV
RWRO@37	3:7		WRO@37+70#PAV
RWRO@46	4:6		WRO@46+70#PAV

**Table 5:** Test results of rejuvenated asphalt.

Property	ASTM Standard	Value for				
		8182	CA3	55	37	46
Softening point/°C	D36	50.4	44.0	51.3	47.8	49.5
Penetration at 25 °C/(0.1 mm)	D5	34.3	80.2	32.1	58.4	35.3

**Table 6:** Common fatigue life evaluation indicators.

References	Evaluation Index	Formula	Notes
Liao [22,23]	N*_f50_*	-	$G_{i}^{*}$—i-th order complex modulus
Cao [26]	N_RG*_	$RG^{*}=\left(G_{i}^{*}-G_{i-1}^{*}\right)/G_{i-1}^{*}$	$\omega_{0}$—initial dissipation energy
Shen [27,28]	N*_1_*	$DER=\left(n\times \omega_{0}\right)/\omega_{i}$	$\omega_{i}$—i-th order dissipation energy
Pronk [29,37]	N*_p_*	$CDER=\sum \omega_{i}/\omega_{i}$	$\omega_{a}$—a-th order dissipation energy
Bonnetti [30]	N*_p20_*	-	$\omega_{b}$—a-th order dissipation energy
Carpenter [31,32]	N*_fm_*	$DR=(\omega_{b}-\omega_{a})/(\omega_{a}*\left(b-a\right))$	a,b,n—loading times

**Table 7:** Fatigue life of rejuvenated asphalt (4.5% strain-control).

Sample	N*_f50_*	N*_1_*	N*_p_*	N*_p20_*	N_RG*_	N*_fm_*
REVO@8182	14,592	8173	12,541	11,080	null	null
REVO@CA3	14,567	9097	12,625	9509	null	null
RWRO@55	27,776	20,907	25,000	22,279	null	null
RWRO@37	21,126	14,186	17,775	17,318	null	null
RWRO@46	26,959	18,167	22,854	20,385	null	null

**Table 8:** Fatigue life of rejuvenated asphalt (5.5% strain-control).

Sample	N*_f50_*	N*_1_*	N*_p_*	N*_p20_*	N_RG*_	N*_fm_*
REVO@8182	9727	8220	7512	7512	null	null
REVO@CA3	9485	8251	9714	7088	null	null
RWRO@55	22,706	16,983	21,103	18,359	null	null
RWRO@37	15,512	13,480	15,968	11,734	null	null
RWRO@46	17,578	13,688	16,205	13,688	null	null

**Table 9:** Fatigue life of rejuvenated asphalt (6.5% strain-control).

Sample	N*_f50_*	N*_1_*	N*_p_*	N*_p20_*	N_RG*_	N*_fm_*
REVO@8182	6856	5671	9025	5191	null	null
REVO@CA3	6723	4516	4198	5110	null	null
RWRO@55	14,148	12,317	16,210	10,941	null	null
RWRO@37	9965	8897	10,606	7981	null	null
RWRO@46	11,557	10,645	11,557	9040	null	null

**Table 10:** Fatigue life change rate at different loading frequencies.

Rejuvenated Asphalt	Change Rate (%) (10 Hz)	Change Rate (%) (15 Hz)
REVO@8182	−66.48	−64.68
REVO@CA3	−65.08	−66.75
RWRO@55	−73.66	−64.58
RWRO@37	−60.60	−67.93
RWRO@46	−49.05	−60.22

**Table 11:** Fatigue life change rate at different loading levels.

Regenerative Asphalt	Change Rate (%) (5.5%)	Change Rate (%) (6.5%)
REVO@8182	−32.20	−53.15
REVO@CA3	−25.46	−46.26
RWRO@55	−17.60	−50.89
RWRO@37	−32.24	−53.92
RWRO@46	−32.85	−55.65

## Data Availability

The data presented in this study are available on request from the corresponding author.
